# Two Long-Acting Antipsychotics in a Patient with Treatment-Resistant Schizophrenia: A Case Report

**DOI:** 10.3390/clinpract15030055

**Published:** 2025-03-10

**Authors:** Salvatore Cipolla, Flora Delli Carpini, Pierluigi Catapano, Valeria De Santis, Antonio Volpicelli, Francesco Perris, Francesco Catapano

**Affiliations:** Department of Psychiatry, University of Campania “Luigi Vanvitelli”, Largo Madonna delle Grazie 1, 80138 Naples, Italy; flora.dellicarpini@gmail.com (F.D.C.); pierluigi.catapano@unicampania.it (P.C.); valeria.desantis@policliniconapoli.it (V.D.S.); antonio.volpicelli@policliniconapoli.it (A.V.); francesco.perris@unicampania.it (F.P.); francesco.catapano@unicampania.it (F.C.)

**Keywords:** long-acting injectable antipsychotics, treatment resistant, schizophrenia, case report

## Abstract

Treatment-resistant schizophrenia (TRS) poses significant therapeutic challenges due to persistent symptoms, poor adherence, and high relapse rates. Long-acting injectable (LAI) antipsychotics offer a promising approach, yet limited evidence exists regarding the combination of two LAI formulations. We report the case of a 62-year-old woman with TRS, characterized by recurrent hospitalizations and inadequate responses to oral and monotherapy treatments. During her latest hospitalization, she received alternating intramuscular administrations of haloperidol decanoate (100 mg/28 days) and aripiprazole (400 mg/28 days). The dual LAI strategy resulted in a marked improvement in psychotic symptoms, functional recovery, and treatment adherence, with no reported side effects. This case highlights the potential benefits of dual LAI therapy in managing TRS, particularly in patients with non-adherence to oral medications or limited response to standard treatments. Additional studies are required to evaluate the long-term effectiveness and safety of this innovative therapeutic approach.

## 1. Introduction

As a severe mental illness, schizophrenia has usually been associated with a progressive impairment in all areas of living, frequent relapses, and a chronic trend. Recent evidence has changed this outlook, since the majority of individuals with schizophrenia can obtain symptom remission and adequate levels of functioning [1]. Schizophrenia treatment remains a substantial challenge due to factors like chronic relapses and varying patient adherence, particularly when non-compliance complicates outcomes. Over the years, new drugs with antipsychotic effects have been developed to treat the different symptoms of schizophrenia and reduce the risk of relapse, and the pharmacological approach is frequently integrated with numerous interventions to increase the possibility of patients’ recovery [2,3,4,5,6]. Schizophrenia is frequently linked to a higher risk of cardiovascular, metabolic, and infectious diseases [7,8,9], largely due to unhealthy lifestyle habits and barriers to accessing screening programs and medical check-ups [10,11]. In our opinion, true recovery is unattainable without a comprehensive and integrated treatment approach [12,13]. Without such an approach, the burden will persist for patients, their families, and the healthcare system, leading to increased hospitalizations, repeated treatment failures, and rising costs associated with ineffective psychosocial interventions. In fact, for patients experiencing acute symptom exacerbation or recurrence of psychosis, the National Institute for Health and Care Excellence (NICE) guidelines recommend treatment with one oral antipsychotic, ideally in conjunction with psychological interventions such as family therapy and individual Cognitive Behavioral Therapy (CBT) provided by a trained therapist [14].

Unfortunately, despite all these attempts to treat the disorder and despite advances in pharmacological treatments, up to 34% of patients with schizophrenia are resistant to treatments, even at the time of first-episode psychosis [15,16,17], experiencing inadequate symptom control, both in terms of positive and negative psychotic symptoms and cognitive and social dysfunction. Recently, the Treatment Response and Resistance in Psychosis (TRRIP) working group was formed to establish consensus criteria to standardize the definition of treatment-resistant schizophrenia (TRS) which can be summarized as the persistence of symptoms despite ≥ 2 trials of antipsychotic medications lasting a minimum of 6 weeks, with a minimum total daily dose equivalent to 600 mg of chlorpromazine (determined using established conversion ratios), with documented adherence [18]. TRS is a prevalent and debilitating condition that significantly impacts patients’ quality of life, posing a substantial economic burden on healthcare systems and contributing to making schizophrenia a highly disabling disease [19,20]. This persistent treatment resistance presents a major challenge for clinicians, who must seek innovative and effective strategies to address the multifaceted nature of the disorder. Achieving better symptom management, enhancing treatment tolerability, and ultimately improving patients’ overall quality of life remain critical goals in the management of TRS. The urgency of developing and implementing new therapeutic approaches is underscored by the growing number of individuals affected by this condition and the limited success of current treatment options.

To treat TRS, clozapine, an atypical antipsychotic drug, is indicated as the drug of choice by the Food and Drug Administration (FDA) [21]. However, up to 60% of patients still do not respond to treatment with clozapine [22,23], and this primarily happens due its adverse effects and the need for frequent blood monitoring, making compliance to this oral treatment an issue for many patients [24,25]. Poor adherence to oral drug treatments is a frequent problem for patients with schizophrenia, representing one of the leading causes of relapse and rehospitalization [26]. Often two antipsychotic drugs are necessary for symptom containment of schizophrenia, both in the acute and maintenance phases [27]. Currently, the evidence supporting the efficacy and safety of oral polypharmacy remains inconsistent [27,28,29,30,31]. Moreover, the prescription of multiple oral antipsychotics has been shown to significantly decrease patient adherence to treatment [28].

Long-acting injectable (LAI) antipsychotics (APs) are concentrated drug formulations designed to release the medication gradually over time following intramuscular administration. These formulations have shown a pharmacodynamic profile comparable to the corresponding oral medication, which ensures the same efficacy and safety. Additionally, they offer the advantage of requiring less frequent administrations (ranging from biweekly to intervals of several months) while maintaining sustained medication levels [32]. Many studies indicate that long-acting injectable antipsychotics improve treatment adherence, enhance clinical outcomes, and contribute to a better quality of life for patients with schizophrenia [33,34,35,36].

Although the use of two LAI APs is not explicitly recommended in current national and international guidelines, this approach is increasingly employed in clinical practice, particularly for patients with TRS [37]. This growing interest in dual LAI therapy reflects its potential as a novel strategy for patients who do not respond to clozapine or those for whom combining oral antipsychotics or adjunctive agents proves ineffective. In fact, the administration of two LAI APs could take advantage of the synergistic effect of the two drugs and their safer mode of administration and could also allow the management of those cases of “pseudo-resistance” due to non-adherence to oral formulations.

While the clinical use of dual LAIs is becoming more common, this practice is underrepresented in the scientific literature, and robust evidence supporting its safety and efficacy is still lacking. Despite this, case reports play a critical role in clinical research by providing valuable, real-world insights into treatment strategies, including patient responses and clinical decision-making processes. Our case report contributes to this body of literature by detailing the use of dual LAI therapy in a patient with a long history of schizophrenia, outlining the rationale behind the decision to use this treatment, and documenting the patient’s clinical outcomes. Our report thus examines the clinical implications, safety, and effectiveness of dual LAI therapy, providing a novel perspective on the utility of polypharmacy in managing long-term schizophrenia, particularly in settings where traditional approaches fall short of therapeutic goals.

This case report has been written following the CAse REport (CARE) guidelines to ensure thoroughness and transparency in the presentation of clinical information [38]. Further details can be found in the CARE checklist, provided as Supplementary Material to this manuscript (Table S1. CARE checklist).

## 2. Case Presentation

A 62-year-old woman, with a history of treatment-resistant paranoid schizophrenia as defined by the DSM-IV and ICD-9 criteria (which remain widely used in clinical practice in Italy, as they are officially adopted by the National Health System), and multiple hospitalizations, was referred to our psychiatric ward in March 2023. Her complex clinical condition at admission was characterized by dysphoric mood, disorganized behavior and hetero-directed aggression, thought disorders such as tangentiality and derailment, delusions of reference that were persecutory and somatic (belief that she was pregnant or seriously ill), auditory hallucinations (multiple male voices dialoguing, commenting, denigrating, and threatening), irregular hypnic pattern, poor personal care, and discontinuity in therapy intake with poor or no insight at all. The patient’s clinical characteristics at admission are synthetized in Table 1.

Although she had already been admitted to a psychiatric hospital four times during the past 8 years, the evidence indicated the progressive course of the disease and her failure to respond to various treatment regimens. The onset of the disease dates to 2000 following the separation from her husband, with the occurrence of isolated persecutory and reference delusions; at that time, the patient and her family did not seek medical help and decided to manage the symptomatology without the aid of medication.

After 4 years, the first worsening of the clinical condition occurred: the delusion of persecution and reference became more impactful on global functioning and auditory hallucinations appeared. In particular, the patient heard the voice of a “mad man” threatening her. Phenomena of theft and influence of thoughts were also present. This time, she was treated with first- and second-generation antipsychotics such as olanzapine and haloperidol. Then, she lived a period of psychiatric well-being with short, pharmacologically managed flare-up phases until 2013. In that year, the patient independently decided to stop taking her medications, so she had a new deterioration, which was managed over the next two years with various pharmacological therapies based on olanzapine, clozapine, olanzapine pamoate injection, and haloperidol decanoate injection up to 150 mg/3 weeks. The use of these therapies did not achieve the desired remission of symptoms; furthermore, olanzapine caused weight gain, clozapine was discontinued due to poor compliance, and antipsychotic treatment resulted in akathisia treated with biperiden. In 2015, the first psychiatric hospitalization was necessary.

During that hospitalization, her symptoms were dysphoric/depressed mood, disorganized behavior and speech, delusion of poisoning, referential and persecutory delusions, auditory hallucinations consisting of several threatening voices, and initial insomnia. Therefore, psychiatrists decided to treat her with haloperidol 50 mg/10 days, escitalopram, biperiden, and benzodiazepines; in the next months, to achieve a greater control of psychotic symptoms and anxiety, trifluoperazine was added.

Over the next 3 years, the patient went through a phase of relative well-being until she was diagnosed with uterine carcinoma in 2018, for which she was surgically treated. After this occurrence, the symptoms flared up again and the patient needed numerous hospitalizations in the psychiatric ward for the management of symptoms and/or the modification of drug treatment. Valproate, risperidone, and paliperidone were prescribed; however, the clinical results were poor. In addition, the need for hospitalizations and the persistence of symptoms contributed to feelings of distrust, reduced compliance with drug treatment, the use of non-pharmacological and unconventional therapies, and a progressive reduction in insight. A chronological overview of the patient’s clinical history and antipsychotic treatments is reported in Table 2.

Given this medical history and her poor compliance with oral medications, during the hospitalization in our psychiatric ward in March 2023, a new therapeutic strategy was developed, and two long-acting antipsychotic formulations were administered. Initially, intramuscular haloperidol decanoate 100 mg/28 days was introduced, as the patient was already receiving intramuscular haloperidol; furthermore, its stronger deliriolytic effect was expected to help manage her symptoms and improve insight. Subsequently, intramuscular aripiprazole 400 mg/28 days was added. It should be noted that the introduction of a second antipsychotic was initially met with reluctance by the patient; however, following a detailed discussion involving the patient, her daughter, and the medical staff, she eventually provided adequate consent for the addition of aripiprazole LAI. The selection of intramuscular haloperidol decanoate and aripiprazole was based on the patient’s documented improvement with these agents during previous treatment phases, as well as on their complementary receptor profiles. This decision was further supported by a careful evaluation of her side-effect history and the need to minimize further non-adherence.

Potential side effects of dual LAI therapy, such as injection site reactions, metabolic disturbances, and extrapyramidal symptoms (EPSs), were anticipated and carefully monitored throughout the treatment period. Injection site reactions—including pain, redness, and local inflammation—were minimized by employing proper injection techniques and rotating injection sites. Notably, the schedule required that one LAI AP be administered every 14 days; consequently, the two drugs were administered alternately to avoid an excessive drug concentration and to reduce the risk of adverse events, pain, and injection site injury. Metabolic disturbances, such as weight gain, dyslipidemia, and hyperglycemia, were regularly assessed through laboratory evaluations (e.g., fasting blood glucose and lipid profiles). Furthermore, any potential onset of EPSs—such as akathisia, rigidity, tremor, and bradykinesia—was monitored daily during psychiatric interviews throughout the entire hospitalization. Patient feedback regarding subjective side effects was also incorporated into our monitoring protocol. This structured approach allowed for the early detection and prompt management of any adverse events, ensuring that the overall safety profile of the dual LAI regimen was maintained throughout the treatment course. In the following weeks, no adverse events appeared, but delorazepam 1.25 mg/day was necessary to treat anxious symptoms and insomnia.

It should be noted that, in addition to pharmacological therapy, during her hospitalization, the patient voluntarily participated in rehabilitation interventions offered to all inpatients. These activities, conducted by trained psychiatric rehabilitation professionals and supervised by medical staff, were tailored to her specific needs using a cognitive behavioral approach. The intervention focused on key topics such as the stress-vulnerability model, early warning signs of relapse, and the importance of adherence to pharmacological treatment. This personalized approach aimed to enhance the patient’s understanding of her condition, promote self-management skills, and support her overall recovery process.

At discharge, after 8 weeks, the patient showed an improvement in symptoms: reduction in the impact of delusions on daily life, reduction in hallucinations, mood improvement, disappearance of disorganized behavior and hetero-directed aggression, and improved hypnic pattern and personal care. The patient’s symptom improvement was evidenced by the reduction in Brief Psychiatric Rating Scale (BPRS) scores, which decreased from a total of 76 at admission to 31 at discharge. At discharge, all items were rated as either 1 (absent) or 2 (very mild), except for the “suspiciousness” item, which scored 3 (mild). The Global Assessment of Functioning (GAF) score also showed notable improvement, rising from an initial 25 (indicating major impairment across all functional areas and behavior influenced by delusions and hallucinations) to 85 (indicating good functioning in all areas, minimal symptoms, and effective socialization). Additionally, trust in medical personnel and adherence to drug treatment also improved significantly: the patient’s insight, assessed using the Insight and Treatment Attitudes Questionnaire (ITAQ), markedly improved from a baseline score of 12 to 20 upon discharge. In the weeks following administration, no side effects were attributable to the pharmacological treatment with two LAI APs.

## 3. Discussion

In the case we described, the patient had been suffering from schizophrenia for more than 20 years and undergone numerous pharmacological therapies, showing a partial clinical response and frequently manifesting poor adherence to medication. Similar to other cases reported in the literature, before considering the dual LAI strategy, an attempt to introduce clozapine had been made, but the patient consistently refused this option [39]. Therefore, given the challenges in adherence among patients with schizophrenia, the choice to initiate dual LAI therapy was primarily driven by the need to ensure sustained treatment engagement and symptom control. The administration of two LAI APs resulted in a clinical reduction in positive and negative symptoms of schizophrenia and a full functional recovery. Importantly, no significant adverse effects were observed during the treatment period, supporting the feasibility of this approach in carefully selected patients.

The decision to combine haloperidol decanoate 100 mg/28 days and aripiprazole 400 mg/28 days was based on multiple factors, including the patient’s prior response to oral haloperidol and the favorable safety and tolerability profile of aripiprazole. Additionally, as described in other reports [40,41], the combination of a first-generation and a second-generation antipsychotic was considered advantageous to leverage their complementary pharmacodynamic effects on different receptor pathways implicated in schizophrenia. In our specific case, treatment selection was further influenced by the fact that the patient had already been receiving haloperidol at the time of admission and had previously demonstrated a robust clinical response to the combination of haloperidol and aripiprazole, with minimal side effects. In contrast, other pharmacological approaches had either yielded suboptimal efficacy or had been associated with significant adverse effects such as sedation or weight gain. The strong deliriolytic properties of haloperidol, combined with the broader receptor modulation profile of aripiprazole, provided additional rationale for selecting this dual LAI regimen.

The two LAI APs were not administered simultaneously. This decision was based on several considerations: (1) the need to test the initial response to haloperidol decanoate alone; (2) the aim to avoid an excessively high peak in drug concentration in the hours following intramuscular injection; and (3) the effort to minimize the risk of pain and injury at the injection site. Notably, the ability to administer each LAI on a once-monthly schedule allowed for injections to be spaced two weeks apart, reducing overall procedural discomfort and enhancing adherence. Furthermore, patient preference played a key role in treatment planning, as she expressed a preference for alternating the administration of the two intramuscular medications.

The involvement of the patient in the decision-making process would seem to be fundamental, and consent should be obtained prior to the administration. Patient involvement in treatment decisions is a cornerstone of effective clinical care, far beyond a mere ethical formality. In the case we presented, this engagement was fostered not only by actively involving the patient in the decision-making process but also by integrating her daughter’s support and conducting daily psychoeducational and rehabilitation sessions—standard practice in our psychiatric ward. This collaborative approach deepened the patient’s understanding of her condition and the rationale behind the chosen therapeutic strategy, ultimately enhancing her commitment to the treatment plan. We believe that such an inclusive, patient-centered approach is essential for improving adherence during hospitalization and for maintaining compliance after discharge, thereby contributing to better long-term clinical outcomes.

Our experience supports the potential benefits of dual LAI therapy for patients resistant to standard treatments or with adherence challenges. Our case is in addition to the sporadic cases present in the literature [37,39], and it contributes to the growing body of evidence that dual LAI therapy not only facilitates symptom control but also promotes significant functional recovery and insight, thereby improving quality of life and social reintegration. To the best of our knowledge, no RCTs have evaluated the efficacy and/or safety of this treatment regimen, and only two retrospective observational clinical trials have been performed [40,41].

Based on our experience, the use of two LAI APs can be considered as effective and safe as the use of a single LAI or the combination of two oral drugs; however, such a schedule should only be administered by experienced clinicians in patients with TRS with little or no adherence to oral therapy.

### Limitation and Future Direction

Despite the positive short-term outcomes observed in our patient, long-term follow-up data are lacking. Unfortunately, the patient relocated shortly after discharge and is no longer under our care, which prevented us from establishing a structured follow-up plan, limiting our ability to assess the durability of the treatment benefits. This represents a significant limitation of our case report, as long-term data on treatment efficacy, safety, and adherence could not be collected. Future longitudinal studies are necessary to evaluate the long-term efficacy and safety of dual LAI therapy and to determine whether its benefits justify its higher cost and potential risks.

Moreover, many questions remain unanswered. Given that LAI APs have a higher cost than their oral counterparts [42], is the cost of two LAI APs for the same patient balanced by the benefits related to the reduction in relapses and rehospitalizations and in terms of quality of life? Is it possible to double the side effects by administering two LAI APs? In low- and middle-income countries—where the availability of LAI APs is limited—is it ethical to destinate two LAI APs to the same patient [43,44]? Since our report does not provide long-term information, can dual LAI AP administration have indications in the maintenance of schizophrenia, or is it an approach to be limited to the management of acute phases? Since many authors believe that LAIs can be prescribed even in the early stages of the disease [26], regardless of non-adherence to oral therapy, is it right to consider double LAI a strategy dedicated only to patients with TRS?

It is hoped that in the future, further clinical studies will be carried out on the subject in order to strengthen this data, answer these questions, and possibly include the indication for the use of double LAI APs in the guidelines for the treatment of patients with schizophrenia or other psychiatric disorders.

## Figures and Tables

**Table 1:** Patient’s characteristics at admission.

Characteristic	Description
Age and gender	62-year-old woman
Diagnosis	Paranoid schizophrenia
Co-morbidities	Celiac disease; cervix cancer; and breast cancer
Clinical features	
*Behavioral domain*	Disorganized behavior and hetero-directed aggression
*Affective domain*	Dysphoric mood and anxiety
*Thought process and content domain*	Tangentiality; derailment; and delusions: referential, persecutory, and somatic (e.g., belief of pregnancy or severe illness)
*Perceptual domain*	Auditory hallucinations: multiple male voices
*Sleep*	Irregular sleep pattern and initial insomnia
*Self-care*	Poor personal care
*Insight*	Poor insight
BPRS 4.0 (total score)	76
*Somatic concern*	7
*Anxiety*	6
*Depression*	3
*Suicidality*	1
*Guilt*	1
*Hostility*	7
*Elevated mood*	1
*Grandiosity*	1
*Suspiciousness*	7
*Hallucination*	6
*Unusual thought content*	7
*Bizarre behavior*	3
*Self-neglect*	2
*Disorientation*	1
*Conceptual disorganization*	4
*Blunted affect*	1
*Emotional withdrawal*	1
*Motor retardation*	1
*Tension*	4
*Uncooperativeness*	2
*Excitement*	3
*Distractibility*	2
*Motor hyperactivity*	4
*Mannerism and posturing*	1
b	25
ITAQ score	12

BPRS: Brief Psychiatric Rating Scale; ITAQ: Insight and Treatment Attitudes Questionnaire. Italics indicate the different domains within the broader category above.

**Table 2:** Timeline of the patient’s clinical history and antipsychotic treatments over the years.

Year/Period	Event	Antipsychotic Prescription	Response
2000	Onset of psychotic symptoms	No medical intervention sought by the patient and her family	
2004	First worsening of clinical condition	Olanzapine 10 mg/die Haloperidol *	Symptom remission and relative well-being
2006	Diagnosis of ductal carcinoma in situ, treated with quadrantectomy and radiotherapy		
August 2013	Discontinuation of medication leading to a new deterioration	Olanzapine pamoate * Haloperidol 4 mg/die	No response; weight gain
November 2013	Transition to a new psychiatrist	Haloperidol decanoate 50 mg/2 weeks	Partial response
October 2014	Worsening of symptoms following the daughter’s relocation abroad	Haloperidol decanoate up to 150 mg/3 weeks	Partial response; akathisia
November 2014	Transition to a new psychiatrist	Clozapine *	Non-adherence due to patient refusal
January 2015	First psychiatric hospitalization—patient enrolled in our Mental Health Service	Haloperidol decanoate 50 mg/2 weeks Trifluoperazine 2 mg/die	Symptom remission
December 2017	Diagnosis of high-grade squamous intraepithelial lesion of the cervix, treated with conization		
January 2019	Discontinuation of medication leading to a new deterioration; second psychiatric hospitalization	Haloperidol decanoate 50 mg/2 weeks Aripiprazole 5 mg/die	Good response
February 2020	Poor compliance and progressive worsening of symptoms	Haloperidol decanoate 50 mg/2 weeks Risperidone up to 2 mg/die	Partial response; dose-dependent daytime sedation and clinically relevant psychomotor retardation
June 2020	Worsening of the COVID-19 pandemic in Italy	Paliperidone palmitate 100 mg/2 weeks	No response
September 2020	Third psychiatric hospitalization	Quetiapine 500 mg/die	Good response
October 2021	Heel fracture and symptom exacerbation; fourth psychiatric hospitalization	Quetiapine 700 mg/die	Partial response
March 2022	Worsening of symptoms	Quetiapine 600 mg/die Trifluoperazine 1 mg/die	Poor response; akathisia and motor stereotypies; and weight gain
May 2022	Worsening of symptoms	Haloperidol decanoate 50 mg/2 weeks	Partial response
November 2022	Missed appointments, symptom exacerbation	Haloperidol decanoate 150 mg/3 weeks Olanzapine 5 mg/die	Partial response; weight gain; and poor compliance
March 2023	Fifth (current) psychiatric hospitalization	Haloperidol decanoate 100 mg/28 days Aripiprazole 400 mg/28 days	Good response

* Reported use, but exact dosage not recorded.

## Data Availability

The data of this study are available from the corresponding author upon reasonable request. Due to privacy regulations and ethical considerations, only de-identified clinical data can be shared.

## Supplementary Materials

The following supporting information can be downloaded at: https://www.mdpi.com/article/10.3390/clinpract15030055/s1; Table S1: CARE checklist.

## Abbreviations

The following abbreviations are used in this manuscript:

AP(s)	Antipsychotic(s)
BPRS	Brief Psychiatric Rating Scale
CBT	Cognitive Behavioral Therapy
FDA	Food and Drug Administration
GAF	Global Assessment of Functioning
ITAQ	Insight and Treatment Attitudes Questionnaire
LAI	Long-acting injectable
NICE	National Institute for Health and Care Excellence
TRRIP	Treatment Response and Resistance in Psychosis
TRS	Treatment-resistant schizophrenia
