# Supplementary material for: Two Long-Acting Antipsychotics in a Patient with Treatment-Resistant Schizophrenia: A Case Report
Source: Clin Pract. 2025 Mar 10;15(3):55. doi: 10.3390/clinpract15030055 (PMC11941564; doi:10.3390/clinpract15030055)
Supplement: Supplementary file 1 [file clinpract-15-00055-s001.zip › clinpract-3476595-supplementary.pdf]

CARE case report guidelines

CARE Checklist of information to include when writing a case report

(cc) BY-NC-ND

Topic	Item	Checklist item description	Reported on Line
Title	1	The diagnosis or intervention of primary focus followed by the words "case report"	2-4
Key Words	2	2 to 5 key words that identify diagnoses or interventions in this case report, including "case report"	23-24
Abstract (no references)	3a	Introduction: What is unique about this case and what does it add to the scientific literature?	12-13
	3b	Main symptoms and/or important clinical findings	13-15
	3c	The main diagnoses, therapeutic interventions, and outcomes	14-17
	3d	Conclusion—What is the main "take-away" lesson(s) from this case?	19-21
Introduction	4	One or two paragraphs summarizing why this case is unique (**may include references**)	96-103
Patient Information	5a	De-identified patient specific information.	112
	5b	Primary concerns and symptoms of the patient.	116-122, Table 1
	5c	Medical, family, and psycho-social history including relevant genetic information	Table 1, Table 2
	5d	Relevant past interventions with outcomes	Table 2
Clinical Findings	6	Describe significant physical examination (PE) and important clinical findings.	Table 1
Timeline	7	Historical and current information from this episode of care organized as a timeline	Table 2
Diagnostic Assessment	8a	Diagnostic testing (such as PE, laboratory testing, imaging, surveys).	113-114
	8b	Diagnostic challenges (such as access to testing, financial, or cultural)	N/A
	8c	Diagnosis (including other diagnoses considered)	113-114
	8d	Prognosis (such as staging in oncology) where applicable	N/A
Therapeutic Intervention	9a	Types of therapeutic intervention (such as pharmacologic, surgical, preventive, self-care)	161
	9b	Administration of therapeutic intervention (such as dosage, strength, duration)	162-164, 178-179
	9c	Changes in therapeutic intervention (with rationale)	169-173
Follow-up and Outcomes	10a	Clinician and patient-assessed outcomes (if available)	200-215
	10b	Important follow-up diagnostic and other test results	213-215
	10c	Intervention adherence and tolerability (How was this assessed?)	174-190
	10d	Adverse and unanticipated events	189-190
Discussion	11a	A scientific discussion of the strengths AND limitations associated with this case report	282-289
	11b	Discussion of the relevant medical literature **with references**.	234-280
	11c	The scientific rationale for any conclusions (including assessment of possible causes)	222-280
	11d	The primary "take-away" lessons of this case report (without references) in a one paragraph conclusion	277-280
Patient Perspective	12	The patient should share their perspective in one to two paragraphs on the treatment(s) they received	257-268
Informed Consent	13	Did the patient give informed consent? Please provide if requested	Yes ☒ No ☐
